# Isolated Convolutional-Neural-Network-Based Deep-Feature Extraction for Brain Tumor Classification Using Shallow Classifier

**DOI:** 10.3390/diagnostics12081793

**Published:** 2022-07-24

**Authors:** Yassir Edrees Almalki, Muhammad Umair Ali, Karam Dad Kallu, Manzar Masud, Amad Zafar, Sharifa Khalid Alduraibi, Muhammad Irfan, Mohammad Abd Alkhalik Basha, Hassan A. Alshamrani, Alaa Khalid Alduraibi, Mervat Aboualkheir

**Affiliations:** 1 Division of Radiology, Department of Internal Medicine, Medical College, Najran University, Najran 61441, Saudi Arabia; yealmalki@nu.edu.sa; 2 Department of Unmanned Vehicle Engineering, Sejong University, Seoul 05006, Korea; umair@sejong.ac.kr; 3 Department of Robotics and Intelligent Machine Engineering (RIME), School of Mechanical and Manufacturing Engineering (SMME), National University of Sciences and Technology (NUST) H−12, Islamabad 44000, Pakistan; karamdad.kallu@smme.nust.edu.pk; 4 Department of Mechanical Engineering, Capital University of Science and Technology (CUST), Islamabad 44000, Pakistan; manzar.masud@cust.edu.pk; 5 Department of Electrical Engineering, The Ibadat International University, Islamabad 54590, Pakistan; 6 Department of Radiology, College of Medicine, Qassim University, Buraidah 52571, Saudi Arabia; salduraibi@qu.edu.sa (S.K.A.); al.alderaibi@qu.edu.sa (A.K.A.); 7 Electrical Engineering Department, College of Engineering, Najran University, Najran 61441, Saudi Arabia; miditta@nu.edu.sa; 8 Radiology Department, Faculty of Human Medicine, Zagazig University, Zagazig 44631, Egypt; maatya@zu.edu.eg; 9 Radiological Sciences Department, College of Applied Medical Sciences, Najran University, Najran 61441, Saudi Arabia; hamalshamrani@nu.edu.sa; 10 Department of Radiology and Medical Imaging, College of Medicine, Taibah University, Madinah 42353, Saudi Arabia; maboualkheir@taibahu.edu.sa

**Keywords:** magnetic resonance imaging (MRI), brain tumor, machine learning

## Abstract

In today’s world, a brain tumor is one of the most serious diseases. If it is detected at an advanced stage, it might lead to a very limited survival rate. Therefore, brain tumor classification is crucial for appropriate therapeutic planning to improve patient life quality. This research investigates a deep-feature-trained brain tumor detection and differentiation model using classical/linear machine learning classifiers (MLCs). In this study, transfer learning is used to obtain deep brain magnetic resonance imaging (MRI) scan features from a constructed convolutional neural network (CNN). First, multiple layers (19, 22, and 25) of isolated CNNs are constructed and trained to evaluate the performance. The developed CNN models are then utilized for training the multiple MLCs by extracting deep features via transfer learning. The available brain MRI datasets are employed to validate the proposed approach. The deep features of pre-trained models are also extracted to evaluate and compare their performance with the proposed approach. The proposed CNN deep-feature-trained support vector machine model yielded higher accuracy than other commonly used pre-trained deep-feature MLC training models. The presented approach detects and distinguishes brain tumors with 98% accuracy. It also has a good classification rate (97.2%) for an unknown dataset not used to train the model. Following extensive testing and analysis, the suggested technique might be helpful in assisting doctors in diagnosing brain tumors.

## 1. Introduction

Brain tumors are one of the most dreaded disorders in medical research. The term “brain tumor” refers to the uncontrolled and abnormal growth of cells within the brain that can affect the brain’s regulating mechanisms [1,2]. Brain tumors can expand and spread, putting pressure on the brain and negatively impacting physical health. In the United States, brain tumors were the leading cause of cancer-related mortality among youngsters (0–14 years old) in 2016 [1]. Therefore, the early identification and categorization of brain tumors is a vital study subject in the medical imaging domain. As a result, it aids in determining the best treatment choice to save a patient’s life [2]. Glioma, meningioma, and pituitary tumors are common brain tumor types. However, the degree of malignancy varies between each type of tumor. For example, the most frequent type of primary malignant brain tumor in the adult is glioma grade IV (glioblastomas), which are the fastest growing tumor and making up more than half of all gliomas [3,4]. Benign meningioma, on the other hand, is a slow growth rate tumor that originates in the region that safeguards the brain and spinal cord (the membrane) and is considered the most common primary benign brain tumor in the adult [5]. Pituitary tumor occurs in the pituitary gland area. It is likewise benign in nature but can cause various medical complications, unlike endocrine disorders [6].

Non-invasive brain magnetic resonance imaging (MRI) is the most often used tool for detecting and tracking the progression of brain tumors. Compared to computed tomography, an MRI image provides precise information on brain anatomy [7]. While examining the numerous MRI slices for patients with brain tumors, radiologists find brain malignancies. However, radiologists are frequently confronted with massive numbers of MRIs and several complicated cases. Therefore, diagnosis and distinguishing different types of brain tumors are challenging tasks. As a result, an automated computer-aided diagnostic strategy can be helpful in assisting radiologists in fast brain abnormality detection.

The convolutional neural network (CNN) is the most modern and extensively utilized deep learning approach in automatic computer-aided medical imaging [8,9,10]. The design of the CNN is inspired by brain anatomy. The nodes of the CNN function similarly to neurons in the brain; they take in data (input), analyze them, and present the results (output) [11,12]. Because of its excellent accuracy, the CNN has already been used in multiple studies to detect tumors in brain MRI images [13,14]. In one study, a CNN was proposed to categorize brain MRI images into three subclasses [15]. To avoid model confusion, the researchers used brain MRI axial images from an online publicly available dataset. A classification accuracy of 91.43% was reported for the proposed model. In another study [16], a three-stage framework was proposed to classify brain MRI scans into multi-grade tumors. The recommended technique includes segmentation, augmentation, and classification using a pre-trained CNN (VGG19). The pre-trained network was fine-tuned and had a classification accuracy of 96.56%. The authors of Ref. [17] proposed a modified pre-trained ResNet-50 network to increase the classification accuracy. The authors added 10 new layers after removing the last five layers of the ResNet-50 network, resulting in an accuracy of 97.2%. A novel CNN model for classifying brain MRI scans into three categories was recently reported [18]. The reported accuracy of the model was 96.56% after performing data augmentation and 10-fold cross-validation. In a recent study [19], an isolated model was formed and re-utilized to classify brain MRI tumor images. Their model showed a validation accuracy of 95.75%. The brain MRI scans were classified using pre-trained GoogLeNet and ResNet-50 networks [17,20]. However, pre-trained networks require a significant amount of training time. Meanwhile, classical machine learning classifiers (MLCs) relied on the most relevant feature of brain MRI scans to detect brain tumors. Therefore, MLCs need less model training time. Recently, the gray-level co-occurrence matrix (GLCM) features were computed for brain MRI images to train the model for classification [21]. Because the brain MRI scans are so comparable, the accuracy of these global-level characteristics is not very good. Therefore, various local-level brain MRI image features such as scale-invariant feature transformation (SIFT), Fisher vector, and bag of words (BoWs) are utilized for classification [22,23,24]. The concatenation of the GLCM, BoW, and histogram intensity characteristics for brain MRI data achieves 91.28% accuracy [25]. The authors used pre-trained CNNs to compute the features of brain MRI images [26]. The findings indicate that the hybrid deep-feature-trained classical classifier had the highest accuracy of 93.72%. However, the training vector size was large, needing a long training time.

Motivated by the need to enhance the dependability, accuracy, early identification, and true brain tumor classification, this paper presents a deep-feature training approach for automated identification and distinction of brain tumors. In this paper, several CNN models (19, 22, and 25 layers) are developed to classify brain MRI images. Following the development of the model, the transfer learning technique is employed to calculate the deep features of the developed CNN model for MLC training to classify the brain MRI images. Finally, the performance of the suggested technique is tested and compared with existing methods reported in the literature using several online available brain MRI datasets. An unknown dataset is also evaluated to ensure the suggested framework’s reliability, applicability, and stability.

The paper layout is as follows: Section 2 describes the proposed framework, Section 3 and Section 4 present the results and discussion, and Section 5 outlines the conclusion.

## 2. Materials and Methods

**Figure 1:**
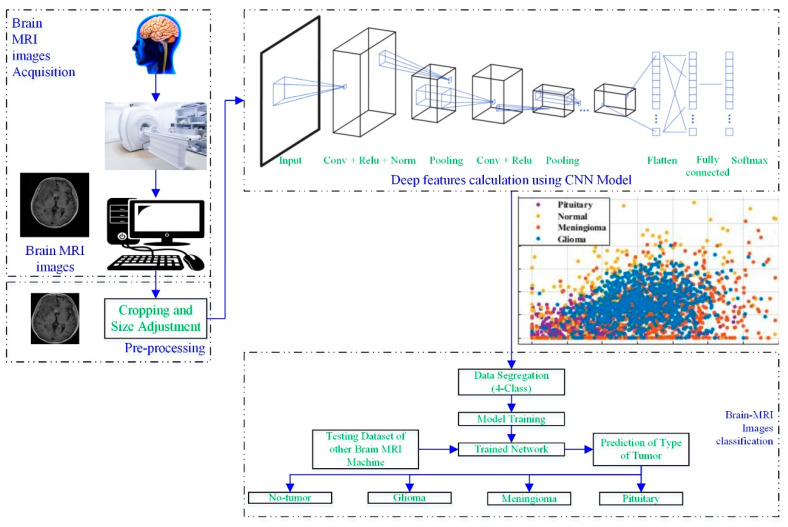
Working framework of the proposed approach.

Figure 1 depicts a process framework for a novel brain MRI-image-based tumor diagnosis and classification approach that employs deep features of the developed-trained CNN.

The brain scans were acquired using a brain MRI machine. Then, the MRI images were pre-processed to adjust the size, according to the CNN. In the next step, the developed CNN models (19 layers, 22 layers, 25 layers) were utilized to extract the deep features of the scans, as shown in Figure 1. Various classifiers (tree [27], support vector machine (SVM) [28,29], naïve Bayes [30], k-nearest neighbor (KNN) [31], ensemble, and neural network (NN)) were used to check the performance of the computed deep features. Further details about each block are discussed in the following sections.

### 2.1. Brain MRI Image Dataset

**Figure 2:**
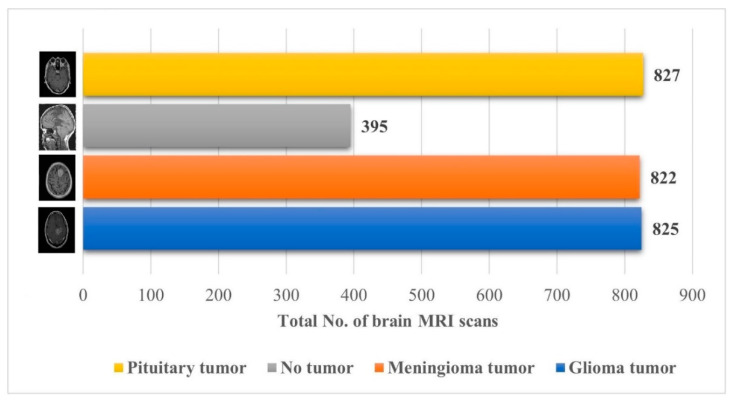
Details about the Kaggle “Brain Tumor Classification (MRI)” dataset [32].

The brain MRI scan dataset for this approach was obtained from the Kaggle “Brain Tumor Classification (MRI)” [32]. The dataset comprises 2870 brain MRI scans with four different types of MRI images; a more detailed description of the dataset is shown in Figure 2.

### 2.2. Pre-Processing

Almost all brain MRI dataset images contain unnecessary spaces and regions, resulting in poor classification accuracy. Therefore, cropping brain MRI scans is essential to eliminate undesired parts (information) and only utilize the image’s valuable information. The cropping method is utilized to compute the extreme point. The noise is eliminated using erosions and dilation; for more information, readers are referred to the reported works [26,33]. The scans were not the same width, height, or dimension; all images were adjusted to 227 × 227 to maintain uniformity.

### 2.3. Deep-Feature Extraction

#### 2.3.1. Isolated CNN Model

**Figure 3:**
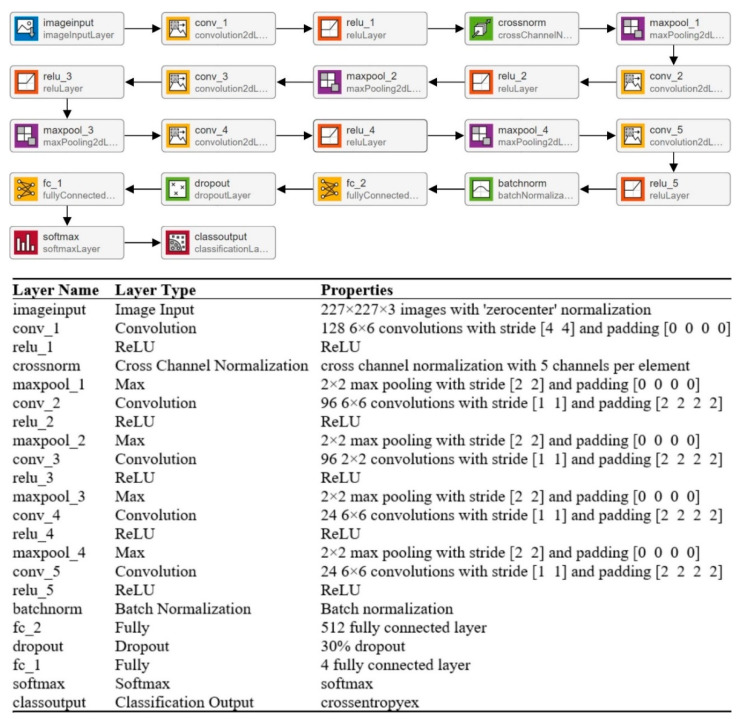
Complete description of the 22-layer CNN model.

CNNs’ strong performance has enhanced their interest among researchers, encouraging them to take on issues previously deemed too difficult to solve. In the last two decades, academics have developed many CNN models to tackle various problems in different sectors, particularly medical imaging [34]. The CNN model’s overall architecture comprises several layers on top of each other. The architecture of the CNN is divided into two parts: feature extraction and classification. Convolutional layers are used to learn the features, while pooling layers are used for image dimensionality reduction. A fully connected (FC) layer is used in a classification module to classify the image. In this paper, multiple layers (19, 22, and 25) of CNN models are trained to categorize MRI scans into multiple categories. The isolated CNN with 22 layers provides the greatest classification accuracy of 92.67%; for further details, readers are referred to a reported work [19]. The complete architecture and description of the 22-layer CNN model are presented in Figure 3.

#### 2.3.2. Feature Extraction Using Developed CNN Model

A machine learning classifier’s performance is largely dependent on an input feature vector. Therefore, designing an algorithm to extract relevant and discriminatory features from brain MRI images is critical for effective tumor classification. In this work, various isolated trained CNNs (19, 22, and 25 layers) were utilized to extract the deep features. The deep features were extracted using the transfer learning method, as discussed in [26,35]. The authors computed the deep features of the pre-trained model for the brain MRI dataset and concatenated various features for better classification performance. In this work, the developed model’s final pooling layer was utilized to extract the deep features of the brain MRI dataset. The detailed specifications of the 22-layer CNN model are shown in Figure 3.

### 2.4. Classification

At this stage, the extracted deep features were fed into renowned classifiers, such as a tree, NN, Naïve Bayes, KNN, ensemble, and SVM. Cortes and Vapnik proposed the SVM classifier in 1992 [36]. SVM is a type of general linear/classical classifier that uses the concept of supervised learning. It increases the margin among the hyperplanes and training features to improve model accuracy and minimize overfitting [37]. In SVM, the high non-linear dataset is mapped into a high-dimensional feature space, and the decision boundaries can be used to segregate them. SVM aims to narrow the gap between the input feature vector and hyperplanes (also called support vectors). For further details about SVM, readers are referred to a reported work [36].

## 3. Results

To run the simulations and complete the analyses, MATLAB 2021a (Natick, MA, USA) was used on a personal computer. Furthermore, to avoid overfitting, the brain MRI dataset was randomly divided into two groups for model training and testing, with a ratio of 0.8 and 0.2, respectively.

**Figure 4:**
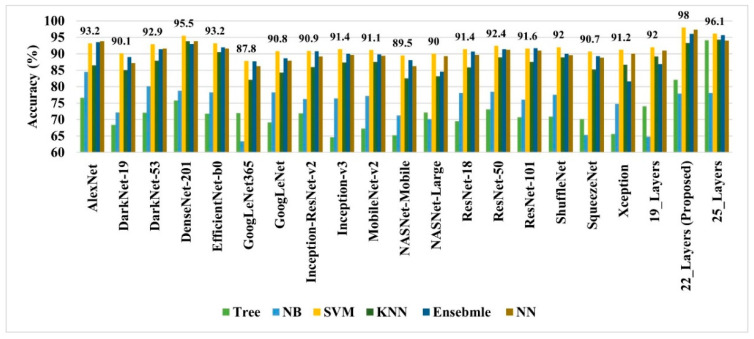
The performance comparison of various deep-feature-trained classical classifier models.

As discussed earlier, the 19-, 22-, and 25-layer isolated CNN models were trained to classify the brain MRI images. The classification accuracies of 91.27%, 92.67%, and 91.62% were found for the four classes (glioma tumor, meningioma tumor, no-tumor, and pituitary tumor) using 19-, 22-, and 25-layer isolated CNN models, respectively. After that, the developed CNN and pre-trained models were used to extract the deep features for classifying brain MRI images using classical/linear classifiers. The classification accuracy was used as a comparison metric for all models, and the results are presented in Figure 4.

**Figure 5:**
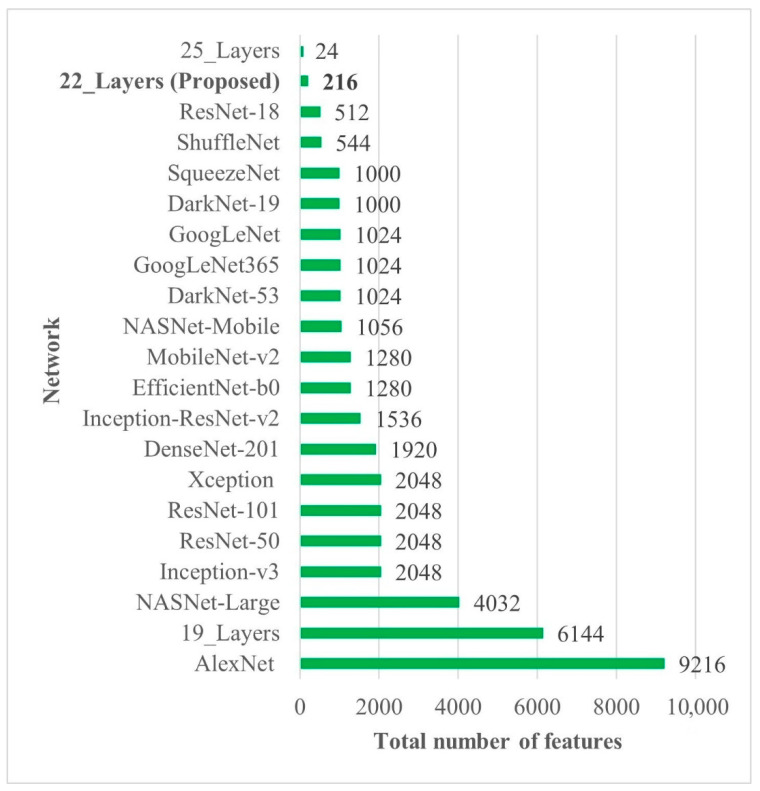
Deep-feature vector size comparison of all CNN models. Bold is used to highlight the best results.

In all the deep-feature-trained models, SVM showed the best accuracy. That is why only the results of the SVM models of all deep-training features are labeled in Figure 4. It is evident from Figure 4 that the deep features extracted from the 22-layer SVM model had the best validation accuracy of 98% for brain tumor classification. The comparison of feature vector size for one brain MRI image is presented in Figure 5.

**Figure 6:**
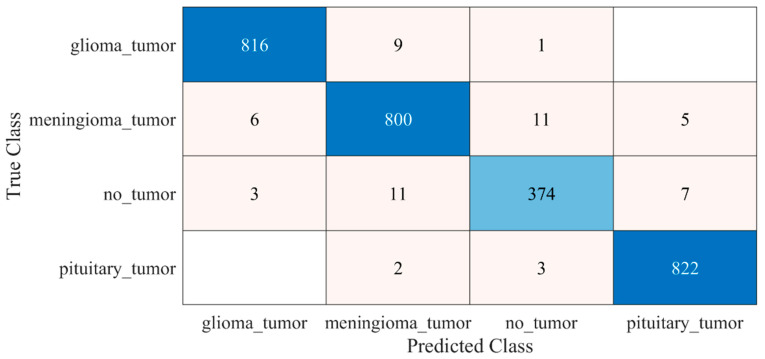
Confusion matrix of proposed deep-feature-trained SVM model. Blue color (dark and light) represent the number of correctly classified samples whereas other colors represent the misclassified samples.

The detailed results of the 22-layer CNN deep-feature-trained SVM model are shown in Figure 6 and Table 1.

**Figure 7:**
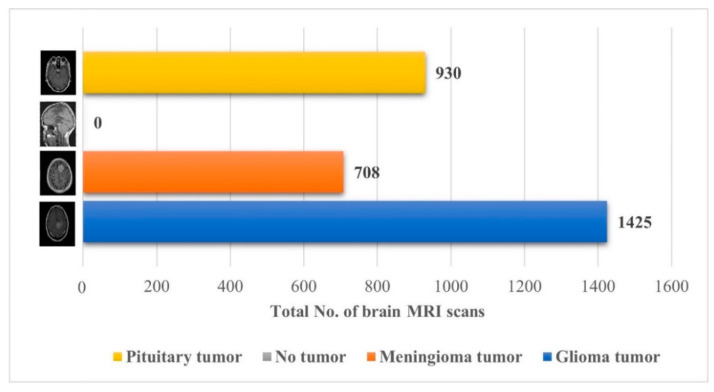
Details about the unseen brain MRI dataset used for testing the proposed model [38].

The performance of the proposed approach was also checked on an unseen dataset [38], which contains the MRI brain images of 233 patients, and the complete description (no. of brain MRI images per class) of the dataset is presented in Figure 7.

**Figure 8:**
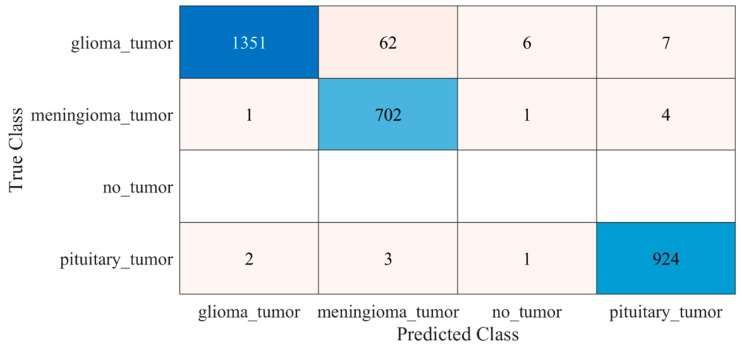
Confusion matrix of the testing of the proposed trained model for an unseen dataset. Blue color (dark and light) represent the number of correctly classified samples whereas other colors represent the misclassified samples.

The proposed trained model was also tested against an unseen dataset to validate the adaptability of the proposed model. The testing results of the model are illustrated in Figure 8 and Table 2.

The proposed model performed well for an unseen brain MRI dataset, which was not used to train the proposed model. It reflects the adaptability and reliability of the proposed approach for real-time applications. The proposed model results are also compared to the state-of-the-art approaches present in the literature. Table 3 compares the proposed tumor diagnosis model to existing techniques based on accuracy.

## 4. Discussion

Recent advances in computer-aided diagnostic technology have made it easier for physicians to diagnose diseases earlier. These developments benefit them in various medical disciplines, particularly in disease detection, treatment, and rapid decision making. Hospitals acquire a tremendous amount of medical data every day. Medical informatics research aids scholars and clinicians in properly utilizing this vast volume of medical data [43].

Early identification and proper treatment choices are required to treat brain tumors effectively. The stage, type, and grade of the tumor at the time of diagnosis determine the best treatment choice. Various methodologies, from pre-trained CNN to developed CNN models, are proposed to detect brain tumors using brain MRIs [19,26,39,40,41,42]. Researchers have also computed the features to train classical classifiers [25]. For high accuracy, the deep learning model needs a long training period and a large training dataset. Three different CNN models (19, 22, and 25 layers) are developed in this work to categorize brain MRI images. The reported accuracies of the models are 91.27%, 92.67%, and 91.62%, with almost 15 min of training time for 19, 22, and 25-layer models, respectively. The model has low accuracy with a long training time. Alanazi et al. [19] re-utilized the trained model (22-layer CNN) using the transfer learning approach to detect the subclasses of tumors. Their proposed approach has an accuracy of 95.75%, as shown in Table 3. Various researchers computed the deep features of the pre-trained model to train the classical classifiers such as SVM [26,42]. With the concatenated training feature vector, the researchers were able to attain an accuracy of 93.72% [26]. The feature vector’s size is large, which necessitated a longer training time. This study provided a novel way to compute the deep features of the developed CNN model to minimize the feature vector size and improve the model’s accuracy. The deep features of the CNN models with 19, 22, and 25 layers were calculated and utilized to train the different models. A 19-layer CNN has a feature vector size of 6144 and a poor accuracy of just 92%, while a 25-layer CNN has a vector size of only 24 features but a lower accuracy than the 22-layer deep-feature-trained model (see Figure 4 and Figure 5). The DenseNet−201 has the highest accuracy of 95.5% in the pre-trained deep-feature-trained model, with a feature vector size of 1920. Therefore, with an appropriate feature vector size (216 features), the 22-layer computed deep-feature-trained SVM model provides the greatest accuracy of 98%. The presented model is additionally evaluated with an unknown dataset that was not utilized for model training to ensure its reliability and adaptability. As illustrated in Figure 7, the unknown dataset solely comprises MRI images of brain tumor patients. The proposed trained model classifies all the brain MRI images with an accuracy of 97.2%. Only 6 out of 708 brain MRI images were incorrectly classified as meningioma tumors (see Figure 8), ensuring a high true positive rate of 99.2% (see Table 2). Similarly, the pituitary tumor class has a true positive rate of 99.4%, with just 6 out of 930 images misclassified. Table 2 demonstrates that the false discovery rate of the no-tumor class was 100% since there was no MRI image of a healthy patient in the dataset. The proposed approach outclasses other state-of-the-art techniques in the literature, as shown in Table 3. The proposed model exhibits a 98% accuracy for the same dataset used for training and a 97.2% accuracy for unseen brain MRI images. It demonstrates the suggested method’s durability and adaptability. It implies that it can be used in clinical practice to diagnose brain tumors in real time.

## 5. Conclusions

The diagnosis of brain tumors is one of the most essential areas of medicine. This paper presents a novel method for detecting and distinguishing brain tumors utilizing brain MRI images. To categorize brain MRI images, three multi-layer CNN models were constructed, although the classification performance of the built CNN was poor. To enhance the classification accuracy, the deep features of the developed CNN models were computed employing transfer learning and used to train the MLCs. Compared to other deep-feature-trained (19-layer, 25-layer, and pre-trained CNN) MLCs, the 22-layer CNN deep-feature-trained SVM had a high accuracy of 98% with an adequate feature vector size. The suggested model’s high testing accuracy of 97.2% for unknown brain MRI datasets makes it a strong contender for aiding clinicians in brain tumor diagnosis.

## Figures and Tables

**Table 1:** Performance of proposed deep-feature-trained SVM model.

Label	True Positive Rate (%)	False Negative Rate (%)	Positive Predictive Value (%)	False Discovery Rate (%)	Training Time (s)	Accuracy (%)
Glioma tumor	98.8	1.2	98.9	1.1	1.665	98
Meningioma tumor	97.3	2.7	97.3	2.7		
No tumor	94.7	5.3	96.1	3.9		
Pituitary tumor	99.4	0.6	98.6	1.4		

**Table 2:** Testing performance of the proposed trained model for an unseen dataset.

Label	True Positive Rate (%)	False Negative Rate (%)	Positive Predictive Value (%)	False Discovery Rate (%)	Accuracy (%)
Glioma tumor	94.7	5.3	99.8	0.2	97.2
Meningioma tumor	99.2	0.8	91.5	8.5	
No tumor	-	-	-	100.0	
Pituitary tumor	99.4	0.6	98.8	1.2	

**Table 3:** Performance comparison of the proposed model with literature.

Study	Approach	Accuracy (%)
Afshar et al. [39]	Capsule network	90.89
Cheng et al. [25]	BoG-trained SVM	91.28
Irmak. [40]	CNN	92.66
Kang et al. [26]	Pre-trained models’ deep-feature-trained SVM	93.72
Alanazi et al. [19]	Developed transfer learned CNN	95.75
Rehman et al. [41]	Pre-trained CNN (AlexNet)	95.86
Ari et al. [42]	Pre-trained models’ deep-feature-trained extreme learning machine	96.88
Proposed Model	Developed CNN model’s deep-feature-trained SVM	98

## Data Availability

Not applicable.
